# Efficacy of Chitosan-N-Arginine Chitosomes in mRNA Delivery and Cell Viability Enhancement

**DOI:** 10.1021/acsabm.4c00983

**Published:** 2024-11-19

**Authors:** Bianca B. M. Garcia, Stefania Douka, Omar Mertins, Enrico Mastrobattista, Sang W. Han

**Affiliations:** † Department of Biophysics, Paulista School of Medicine, Federal University of São Paulo, 04023-062 São Paulo, Brazil; ‡ Pharmaceutics Division, Utrecht Institute for Pharmaceutical Sciences (UIPS), Faculty of Science, Utrecht University, Universiteitsweg 99, 3584 CG Utrecht, The Netherlands

**Keywords:** Chitosan, Lipids, Hybrid systems, Chitosome, mRNA delivery, Gene delivery

## Abstract

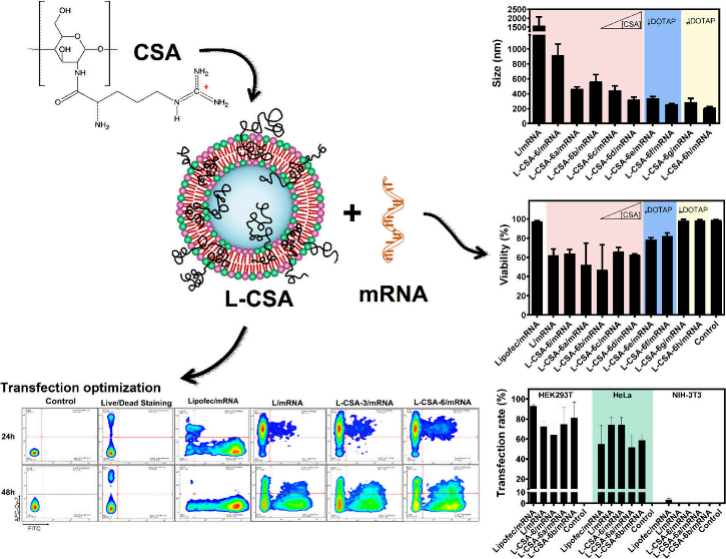

Cationic lipid-based carriers are recognized for their ability to complex with mRNA and effectively deliver the mRNA for vaccination and therapeutic purposes. However, the significant cytotoxicity of these carriers often restricts their practical application. In the present study, polymer–lipid hybrid nanoparticles, termed chitosomes, incorporating chitosan-N-arginine (CSA) with the DOTAP cationic lipid and the DOPE helper lipid, were synthesized and evaluated. The addition of CSA to the lipid formulations improved their physicochemical stability and enhanced mRNA complexation, resulting in high transfection rates in the HeLa and HEK293T cell lines. However, the transfection efficiency was low in the NIH-3T3 cell line, indicating a cell type-specific response to chitosomes. Importantly, CSA significantly reduced the cytotoxicity typically associated with DOTAP. Overall, the present study indicated that optimizing the ratio of CSA to DOTAP is crucial for developing mRNA nanocarriers to achieve high transfection efficiency and reduce cytotoxicity across different cell lines.

## 1 Introduction

mRNA has become a potent instrument for protein replacement,^1^ genome editing,^2^ vaccines,^3,4^ and immunotherapies^5^ due to its high transfection efficiency, lack of integration into the host genome, and lack of risk of infection. The use of mRNA was highlighted in 2020 during the COVID-19 pandemic with the rapid development of two mRNA-based vaccines made by BioNTech/Pfizer and Moderna, which demonstrated greater efficacy (>90%) than other forms of vaccination.^6,7^ This achievement resulted in Katalin Karikó and Drew Weissman receiving the 2023 Nobel Prize in Physiology or Medicine for their pioneering contributions, which fundamentally altered the comprehension of how mRNAs engage with the immune system, enabling the development of highly effective mRNA vaccines against COVID-19.^8^

Compared with plasmid DNA (pDNA) for gene therapy and genetic vaccines, mRNAs offer significant advantages by enabling faster protein expression, as they bypass the nuclear barrier and avoid the potential risk of vector integration within the genome.^9,10^ Nevertheless, the success of mRNA therapy depends on the efficacy and safety of the carrier in safeguarding the mRNA from RNases and ensuring its effective delivery to the targeted cells.^11,12^

Many studies have been performed with the aim of developing efficient nonviral transfection platforms to enhance gene delivery. Cationic lipid-based vectors are among the most popular nonviral gene carriers because of their affinity for nucleic acids via electrostatic interactions and their ability to facilitate efficient internalization and endosomal escape.^13−15^ However, the significant cytotoxicity of cationic lipid-based vectors remains a substantial obstacle in gene delivery applications, necessitating the development of alternative strategies to reduce these adverse effects. Hybrid systems, such as P-LNPs, which combine lipids with polymers, represent a promising approach for gene delivery because they capitalize on the advantageous delivery mechanisms of cationic lipids while minimizing their cytotoxicity through their association with biocompatible polymers.^16,17^

Chitosan (CS) is a promising candidate in the development and utilization of hybrid delivery systems, owing to its wide range of properties, including biological system compatibility, biodegradability, and high efficiency in encapsulating therapeutic agents.^18−21^ Although CS has numerous benefits, its limited solubility in water at physiological pH represents a significant obstacle to its use as a carrier for genetic material; therefore, researchers have explored various chemical modifications to overcome this limitation and broaden its application scope. Examples of such modifications include the utilization of chitosan coated with hyaluronic acid (HA),^22^ the incorporation of poly(2-propylacrylic acid (PPAA),^23^ and the modification of CS with arginine (CSA).^20,24^ These endeavors are geared toward enhancing colloidal stability, hemocompatibility, and/or transfection efficiency, thereby facilitating the development of more effective and versatile delivery systems.

CSA has several distinguishing benefits over other chitosan-based systems that have been previously published. Arginine peptides have a guanidinium group (p*K*_a_ = 13) that allows them to acquire a positive charge in biological media. Arginine also improves the efficacy of CS, owing to rapid internalization by inducing active micropinocytosis.^20,24−27^ We have previously investigated the use of CSA in the production of chitosomes, demonstrating high effectiveness in pDNA delivery.^21^ The present study evaluated the suitability of chitosomes for effective and nontoxic delivery of mRNA. Various chitosomes were prepared with varying amounts of CSA and cationic lipids to evaluate the role of CSA in stability and transfection efficiency in HEK293T, NIH-3T3, and HeLa cells. The present study contributes to improving the design of innovative nanohybrid systems using lipids and chitosan derivatives for mRNA delivery, thereby offering new insights and approaches that can help advance gene delivery research.

## 2 Experimental Section

### 2.1 Materials

The 1,2-dioleoyl-*sn*-glycero-3-phosphoethanolamine (DOPE) and 1,2-dioleoyl-3-trimethylammonium-propane (DOTAP) lipids were purchased from Avanti Polar Lipids (Alabama, USA). Dulbecco’s modified Eagle’s medium (DMEM) low glucose, DMEM high glucose, sterile phosphate-buffered saline (PBS), fetal bovine serum (FBS), nuclease-free water, trypsin, and agarose were purchased from Sigma–Aldrich (St. Louis, USA). Lipofectamine 3000, Hoechst 33342 (2′-[4-ethoxyphenyl]-5-[4-methyl-1-piperazinyl]-2,5′-bi-1H-benzimidazole-trihydrochloride-trihydrate), Orange DNA dye, and GeneRuler 1 kb were purchased from Thermo Fisher Scientific (Waltham, USA). Zombie NIR viability stain was purchased from BioLegend (Uithoorn, The Netherlands). CleanCap eGFP-mRNA (5 moU) was purchased from Trilink BioTechnologies (San Diego, USA). Midori Green Advance was purchased from Nippon Genetics Europe (Düren, Germany). Water was purified, deionized to 18.2 MΩ cm resistivity, and filtered (0.22 μm). All the chemicals were of analytical grade.

### 2.2 Chitosomes and Chitosome/mRNA Preparation

CSA samples with different degrees of arginine substitution (CSA-3 and CSA-6) were synthesized and characterized following the procedure described by Garcia et al.^28^

NPs were prepared using the reversed-phase evaporation technique, based on a previously reported method^29,30^ with some modifications. Briefly, an aliquot of CSA aqueous dispersion, which had been previously prepared in Milli-Q water and agitated overnight, was added to a 15 mL Falcon tube containing presolubilized lipids DOPE:DOTAP 1:1 (w/w) in chloroform. The mixture was vortexed for 1 min to form a water–oil emulsion, after which the solvents were evaporated using nitrogen gas injection. The resulting NPs were suspended in nuclease-free water, with a final volume of 5 mL for L, L-CSA-3, and L-CSA-6, and 1 mL for the other formulations. Comprehensive details of the preparation are provided in Supplementary Tables S1 and S2. The suspensions were vortexed for 2 min and sonicated using an ultrasonic tip probe sonicator (Φ 3 mm, 40% amplitude, 3 min). The NPs were sterilized by filtration through a 0.22 μm filter. Liposomes (L) were prepared using the same protocol, but without CSA. The chitosome L-CSA-3 and L-CSA-6 were synthesized with CSA-3 and CSA-6, respectively, and the variants of L-CSA-6 (L-CSA-6a, L-CSA-6b, L-CSA-6c, L-CSA-6d, L-CSA-6e, L-CSA-6f, L-CSA-6g, and L-CSA-6h) were also prepared. Table 1 summarizes the final concentration of all components used in the synthesis. All NPs were stored at 4 °C until use.

**Table 1:** Final Concentration of DOPE, DOTAP, and CSA in Liposome (L) and Chitosome (L-CSA) Formulations

NP name	L	L-CSA-3	L-CSA-6	L-CSA-6a	L-CSA-6b	L-CSA-6c	L-CSA-6d	L-CSA-6e	L-CSA-6f	L-CSA-6g	L-CSA-6h
[DOPE] mg mL^–1^	1	1	1	1	1	1	1	1	1	1	1
[DOTAP] mg mL^–1^	1	1	1	1	1	1	1	0.5	0.5	0.1	0.1
[CSA] mg mL^–1^	0	0.04	0.04	0.5	1	2	4	2	4	2	4

EGFP-mRNA (Trilink, USA) was stored at −80 °C as stock or −20 °C for working use until needed. For NP/mRNA complex preparation, nuclease-free water, NP suspension, and mRNA were sequentially pipetted into an Eppendorf tube and mixed by manual pipetting. The NP/mRNA complexes were prepared based on the weight ratio, using the mass of lipids (DOPE:DOTAP) and mRNA. Detailed instructions are provided in Supplementary Table S3, with specific concentrations outlined for each experiment. The NP/mRNA mixture was incubated at room temperature for 30 min to facilitate complex formation before further use.

### 2.3 Characterization

#### 2.3.1 Gel Retardation Assay

The complexation of chitosomes/mRNAs was assessed via agarose gel electrophoresis. The samples were prepared at NP/mRNA weight ratios of 4:1, 8:1, and 16:1, as previously described (2.2). The total volume of each sample was adjusted to 10 μL, with 150 ng of mRNA remaining per sample, unless otherwise specified. Subsequently, 6x-Orange DNA loading dye was added to each sample for nucleic acid staining, and the samples were loaded into a 1% agarose gel in Tris-acetate-EDTA (TAE) buffer containing 5 μL of Midori Green. Electrophoresis was performed at 80 V for 40 min. The agarose gel was analyzed using the ChemiDoc Imager (Bio-Rad Laboratories Inc., Hercules, CA) with Image Lab software (version 6.0.1).

#### 2.3.2 Determination of Size and Zeta Potential

The hydrodynamic diameters and PDIs of the chitosomes and the chitosome/mRNA complexes were measured via DLS using a Zetasizer Nano-S at a 90° angle (Malvern Panalytical, Malvern, UK). The zeta potential was determined via a Zetasizer Nano-Z (Malvern Panalytical, Malvern, UK) with universal ZEN 1002 dip cells. For analysis of NP/mRNA complexes, 100 μL of each complex was diluted in 500 μL of 1x PBS. All the measurements were performed at 25 °C, and the data were analyzed via software provided by Malvern.

### 2.4 Cell Culture and *In Vitro* Experiments

HEK293T cells were cultured in low-glucose DMEM supplemented with 10% FBS (v/v). NIH-3T3 and HeLa cells were cultured in high-glucose DMEM supplemented with 10% FBS. All the cell lines were maintained in culture flasks at 37 °C in a humidified incubator containing 5% CO_2_.

#### 2.4.1 mRNA Transfection Assay

One day prior to transfection, the cells were seeded at 12,500 cells/well in flat bottom 96-well plates (black and clear for confocal microscopy and flow cytometry, respectively). The plates were incubated at 37 °C in 5% CO_2_ for 24 h. For transfection, the NP/mRNA complexes were prepared as described in Section 2.2 and allowed to incubate at room temperature for 30 min before being applied to the cells. The cells were then incubated with complexes containing either 150 or 250 ng of mRNA per well and maintained at 37 °C with 5% CO_2_ in a humidified incubator. To evaluate the effect of the incubation time of NP/mRNA in cells for transfection, the transfection efficiency was assessed by changing the medium at either 2 or 6 h following NP/mRNA incubation. For flow cytometry and confocal fluorescence microscopy analysis, the cells were prepared 24 or 48 h after transfection. Lipofectamine 3000 was used as the positive control, following the manufacturer’s protocol, and the same amount of mRNA used in the NP/mRNA complexes was used.

#### 2.4.2 Confocal Microscopy

Qualitative analysis of the transfected cells was performed via assessment of green fluorescent protein (GFP) expression in different cell lines using fluorescence confocal microscopy. After transfection, the cells were washed once with sterile 1x PBS (pH 7.4) and stained with Hoechst 33342 (1:1000 in Opti-MEM) in the dark at 37 °C for 10 min. Subsequently, images were acquired using a Yokogawa Cell Voyager CV700s confocal fluorescence microscope (Tokyo, Japan) and analyzed via ImageJ software.

#### 2.4.3 Flow Cytometry

Transfection efficiency, mean fluorescence intensity (MFI), and cell viability were quantified via flow cytometry. After transfection, the cells were trypsinized, neutralized with medium containing FBS, transferred to a U-bottom 96-well plate, and centrifuged. The cells were then washed once with 1x PBS and labeled with Zombie NIR Fixable Viability dye, following the manufacturer’s protocol. After staining, the cells were washed with 0.5% BSA in PBS (w/v) and fixed with 1% PFA. Nontransfected cells, cells transfected with Lipofectamine 3000, and dead cells were used as control groups. Data acquisition was performed using a FACSCanto II flow cytometer (BD, USA), and the acquired data were analyzed by Flowlogic software (Inivai Technologies, Melbourne, Australia). The detailed parameters for this analysis are provided in Figure S1.

### 2.5 Statistical Analysis

The data were obtained from at least two replicates for each data set, and all the data are presented as the means ± standard deviations. Statistical analyses were performed via GraphPad Prism 6 software. The statistical significance of differences between two groups was determined by Student’s *t* test, and the statistical significance of differences among three or more groups was determined by one-way analysis of variance (ANOVA) with post hoc Tukey’s test, unless otherwise stated. Differences between conditions were considered significant if *p* < 0.05 (*).

## 3 Results and Discussion

### 3.1 Enhancing mRNA Delivery: Optimization of Chitosome-Mediated Transfection in Diverse Cell Lines

To assess the efficacy of chitosomes for mRNA delivery in diverse cell lines and to identify NP attributes and conditions that increase transfection efficiency, initial screening of formulations was performed in HEK293T cells, and the optimal formulations were validated in NIH-3T3 and HeLa cells.

#### 3.1.1 Investigating the NP:mRNA Ratio, Incubation Time, and Cytotoxicity in HEK293T Cells

**Figure 1:**
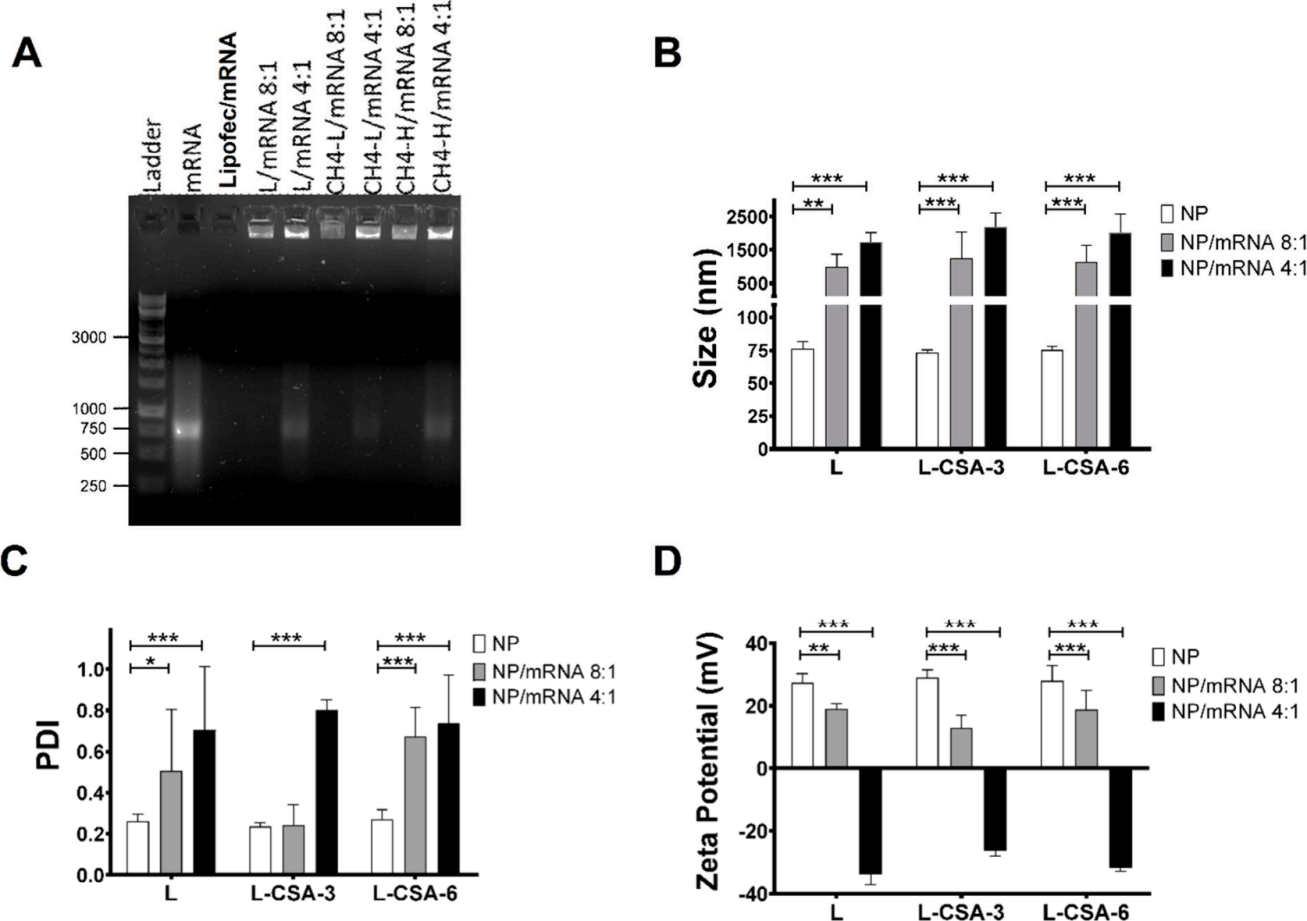
NP/mRNA interaction analyses. (A) The complexation of L, L-CSA-3, and L-CSA-6 with mRNA (150 ng) at ratios of 8:1 and 4:1 was analyzed by agarose gel electrophoresis. The GeneRuler 1 kb DNA ladder was utilized as a molecular weight marker. (B) The size, (C) PDI, and (D) zeta potential of these complexes were determined. Two-way ANOVA was used to compare the differences between the NP and NP/mRNA groups. *p < 0.05, **p < 0.01, and ***p < 0.001.

To evaluate the capacity of different NPs to form complexes with mRNAs, the nanocomplexes were characterized via agarose gel electrophoresis, DLS, and zeta potential measurements. The electrophoresis experiments revealed that at a 4:1 NP to mRNA ratio, liposomes (L) and all the chitosomes partially inhibited mRNA migration, whereas at an 8:1 ratio, complete inhibition was observed, indicating effective interaction and complex formation between the chitosomes and mRNAs (Figure 1A).

In PBS, the NPs exhibited a size range of 73–77 nm, PDI of 0.23–0.27, and zeta potential of 27–29 mV (Figure 1B–D). mRNA complexation resulted in an increase in size and PDI, as well as a decrease in charge, which supported the formation of the mRNA/NP complex (Figure 1B–D). The slightly larger size and PDI values, along with the negative zeta potential of the NP/mRNA complex at a 4:1 ratio compared with the 8:1 ratio, suggest that some mRNA may have remained loosely bound on the NPs surfaces, with mRNA chains extending into solution.

**Figure 2:**
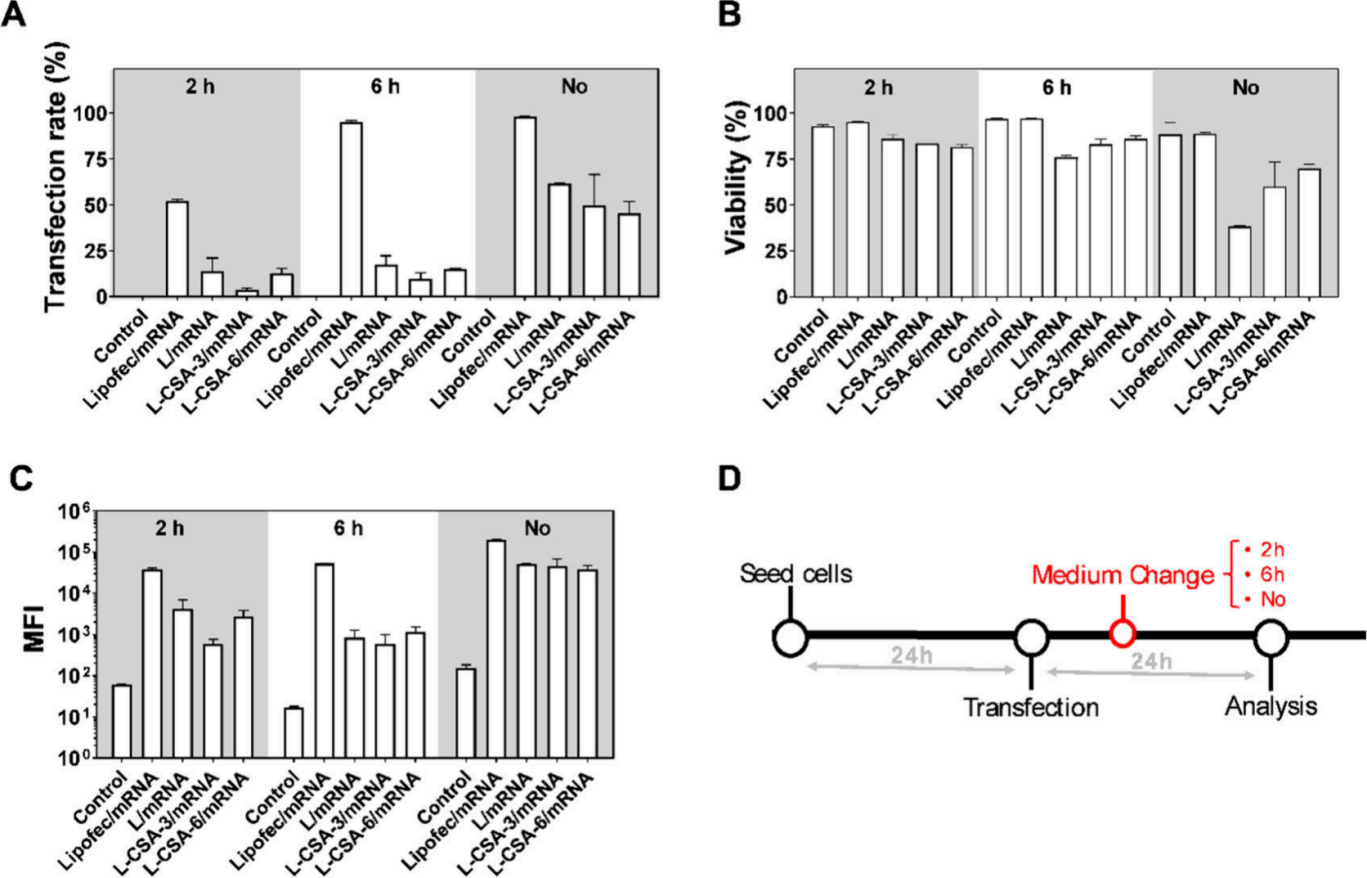
Effect of medium replacement on the chitosome/mRNA transfection efficiency. HEK293T cells were transfected with complexes of mRNA (150 ng per well) with lipofectamine, L, L-CSA-3, or L-CSA-6 at a 8:1 ratio. Analysis was performed 24 h post-transfection, and the effects of medium replacement were compared at 2 h, 6 h, or no medium replacement after complex incubation. The flow cytometry results are presented as the (A) percentage of the transfection rate, (B) cell viability, and (C) mean fluorescent intensity (MFI). Nontransfected cells were used as controls. Details of the gating strategy are shown in Figure S1. (D) Experimental procedure timeline.

Considering the ability of the NPs to complex with mRNA, the transfection efficiency of the L/mRNA, L-CSA-3/mRNA, and L-CSA-6/mRNA complexes at an 8:1 ratio was investigated. The NP/mRNA complexes were prepared with 150 ng of mRNA per well containing 12,500 cells. Transfection efficiency was analyzed 24 h after incubation of NP/pDNA with cells via fluorescence microscopy to identify the transfected GFP^+^ cells. The transfected cells were quantified via flow cytometry (Figure 2). Additionally, the effect of changing the medium at either 2 or 6 h after NP/mRNA incubation was evaluated.

The efficiency of transfection using liposomes and chitosomes did not significantly differ, ranging from 10.0% ± 5.6% with medium replacement 2 h after transfection to 13.9% ± 4.0% with replacement at 6 h and reaching 52.2% ± 8.3% without medium replacement. In contrast, Lipofectamine achieved efficiencies of 52.3% ± 0.6%, 95.0% ± 0.8%, and 98.2% ± 0.2% under the same conditions. The MFI for both liposomes and chitosomes was significantly greater than that of the control, by factors of 41.6 and 49.5, respectively, when the medium was replaced at 2 and 6 h post-transfection, reaching a factor of 291.9 without medium replacement.

HEK-293T cell viability post-transfection was also assessed under those conditions. When the medium was replaced 2 h after transfection, cell viability remained high across all groups: 92.8% for the control, 95.3% for Lipofectamine, 86.1% for L/mRNA, 83.5% for L-CSA-3/mRNA and 81.6% for L-CSA-6/mRNA. With medium replacement at 6 h post-transfection, cell viability remained high in the control and Lipofectamine groups (96.9% and 97.2%, respectively), while L/mRNA viability decreased to 76.0%, and L-CSA-3/mRNA and L-CSA-6/mRNA maintained slightly higher viabilities of 83.0% and 86.0%, respectively. Notably, without medium replacement, cell viability declined sharply, particularly in the L/mRNA group, which dropped to 38.2%. Under these conditions, the chitosome-based formulations L-CSA-3/mRNA and L-CSA-6/mRNA showed improved cell viability at 60.1% and 68.8%, respectively, while the control group and Lipofectamine showed viability of 88.3% and 88.8%, respectively.

These results suggested that replacing the medium before 6 h is crucial for maintaining cell viability but significantly reduces the transfection efficiency. In contrast, the efficiency of Lipofectamine peaked after 6 h (Figure 2).

Although the transfection efficiency in HEK293T cells was lower compared to Lipofectamine,^31,32^ the chitosome formulations still achieved significant transfection efficiency, exceeding 50% in HEK293T cells after 24 h. This suggests that chitosomes complexed with mRNA are relatively safe for use in this cell type. Notably, while L/mRNA complexes showed higher transfection efficiency, the reduced cytotoxicity observed with chitosomes suggests that they may offer a safer alternative for certain applications, despite the slightly lower transfection rates.

#### 3.1.2 The mRNA Concentration and Incubation Time Are Critical for Both the Transfection Efficiency and Cell Viability

**Figure 3:**
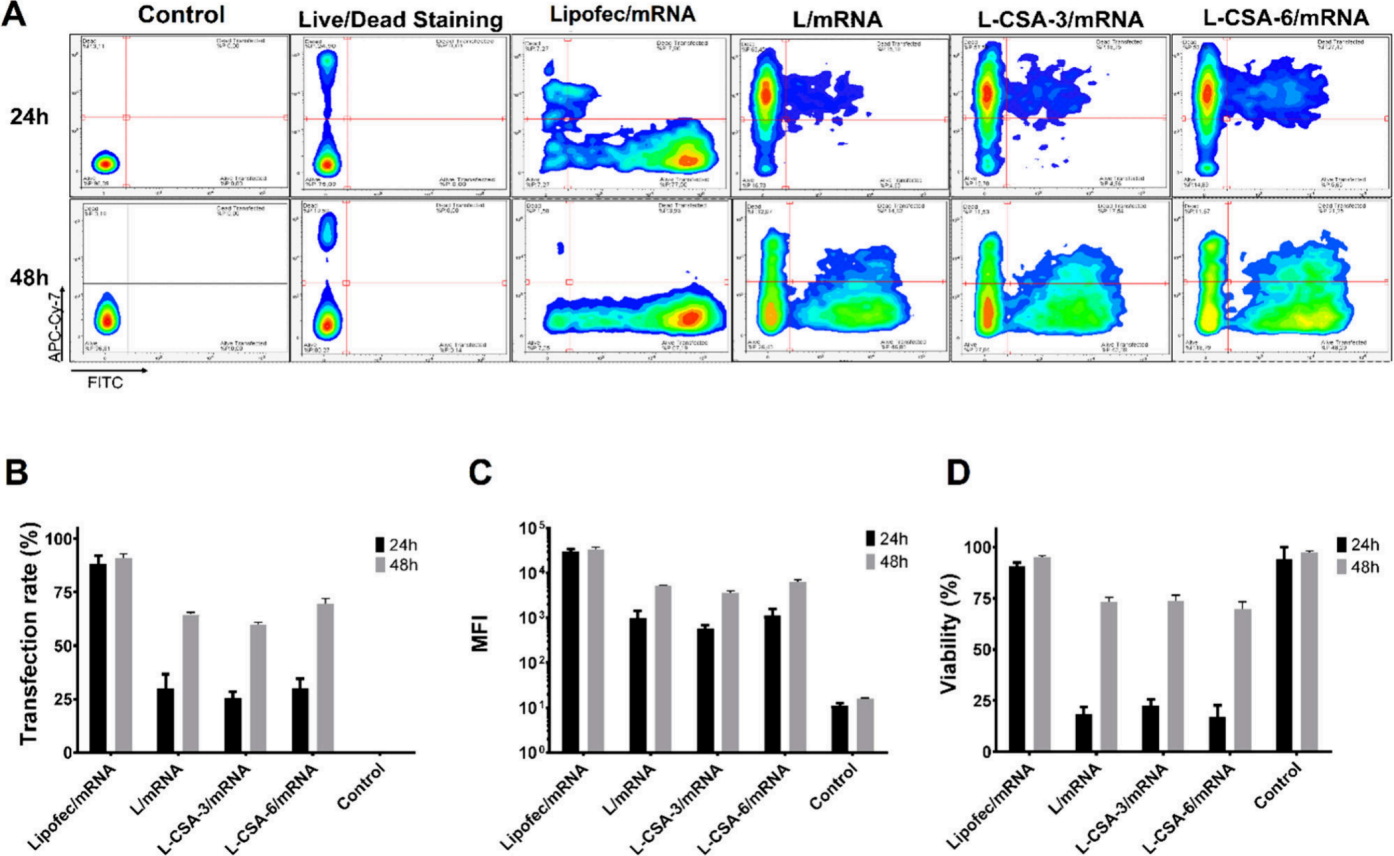
Effect of incubation time on the transfection efficiency of the chitosome/mRNA mixture. HEK293T cells were transfected with mRNA (250 ng per well) complexed with Lipofectamine, L, L-CSA-3, or L-CSA-6, maintaining an 8:1 ratio. The transfection efficiency was assessed at 24 and 48 h post-transfection. (A) Flow cytometry analysis was performed with APC-Cy-7 (ZOMBIE NIR) versus FITC (eGFP) to assess (B) the transfection rate, (C) the mean fluorescence intensity (MFI), and (D) the percentage of live–dead stained cells. Details of the gating strategy are available in Figure S1.

To assess the influence of the quantity of the NP/mRNA complex on the transfection efficiency and establish the optimal timing for analysis, an 8:1 NP/mRNA ratio was used, but the amount of mRNA was increased from 150 ng to 250 ng per well, with 12,500 cells per well. Flow cytometry analysis was conducted 24 and 48 h postincubation of HEK293T cells with the NP/mRNA complexes (Figure 3).

The data from 24 h post-transfection revealed a significant decrease in both the transfection rate and MFI when the amount of NP/mRNA complexes increased from 150 ng to 250 ng of mRNA per well (Figures 2 and 3). At 24 h post-transfection, a significant proportion of cells exhibited positive staining in the live/dead assay, which decreased by 48 h post-transfection (Figure 3D). Furthermore, an extension of the analysis period was associated with an increase in both the transfection efficiency and MFI, which were comparable with those of Lipofectamine.

The cytometry results (Figure 3A) revealed a decrease in the APC-Cy7-positive cell population (indicative of dead cells) 48 h post-transfection. Zombie NIR dye, an amine-reactive fluorescent dye, reacts with free amines in the cytosol, producing red fluorescence (APC-Cy7-positive population). Thus, cells with compromised membranes, typically dead cells, allow the dye to enter, leading to increased fluorescence.^33^ Conversely, the dye interacts primarily with membrane proteins in live cells, resulting in much lower fluorescence levels, thereby facilitating the differentiation between live and dead cells. Increased cell permeability, a precursor to cell death, can also be triggered by external factors, leading to staining with Zombie NIR dye. These results suggested that the binding of liposomes or chitosomes on the cell surface can compromise cell membrane integrity, causing a significant proportion of cells to be stained by Zombie NIR dye. However, cells have the capacity to repair their membranes, a process that can follow the damage caused by transfection. The speed and efficiency of this type of repair depend on the extent of damage and the intrinsic membrane repair mechanisms of the cell.^34,35^ The present study suggested that chitosan caused temporary membrane permeability in HEK293T cells, but this effect was reversible, as evidenced by the recovery of the cells at 48 h post-transfection. These findings highlighted the necessity of carefully balancing the timing of analysis and the quantity of chitosomes/mRNAs to minimize membrane damage. Such optimization helps in cell recovery and maintains viability post-transfection.

### 3.2 CSA Influences Nanoparticle Size and Nanoparticle/Mrna Complexation, Whereas DOTAP Is Responsible for Transfection Efficiency and Cytotoxicity in Chitosomes

To more thoroughly investigate the influence of CSA on cytotoxicity and mRNA delivery efficiency, eight different nanoparticles, each with a unique blend of DOPE, DOTAP, and CSA, were synthesized, and their efficacy in delivering mRNA to HEK293T cells was assessed. For these novel formulations, only CSA-6 was utilized. The final concentration of each component in both liposomes and chitosomes (L and L-CSA-6a-h) is presented in Table 1, and detailed information about their preparation is available in Table S2.

**Figure 4:**
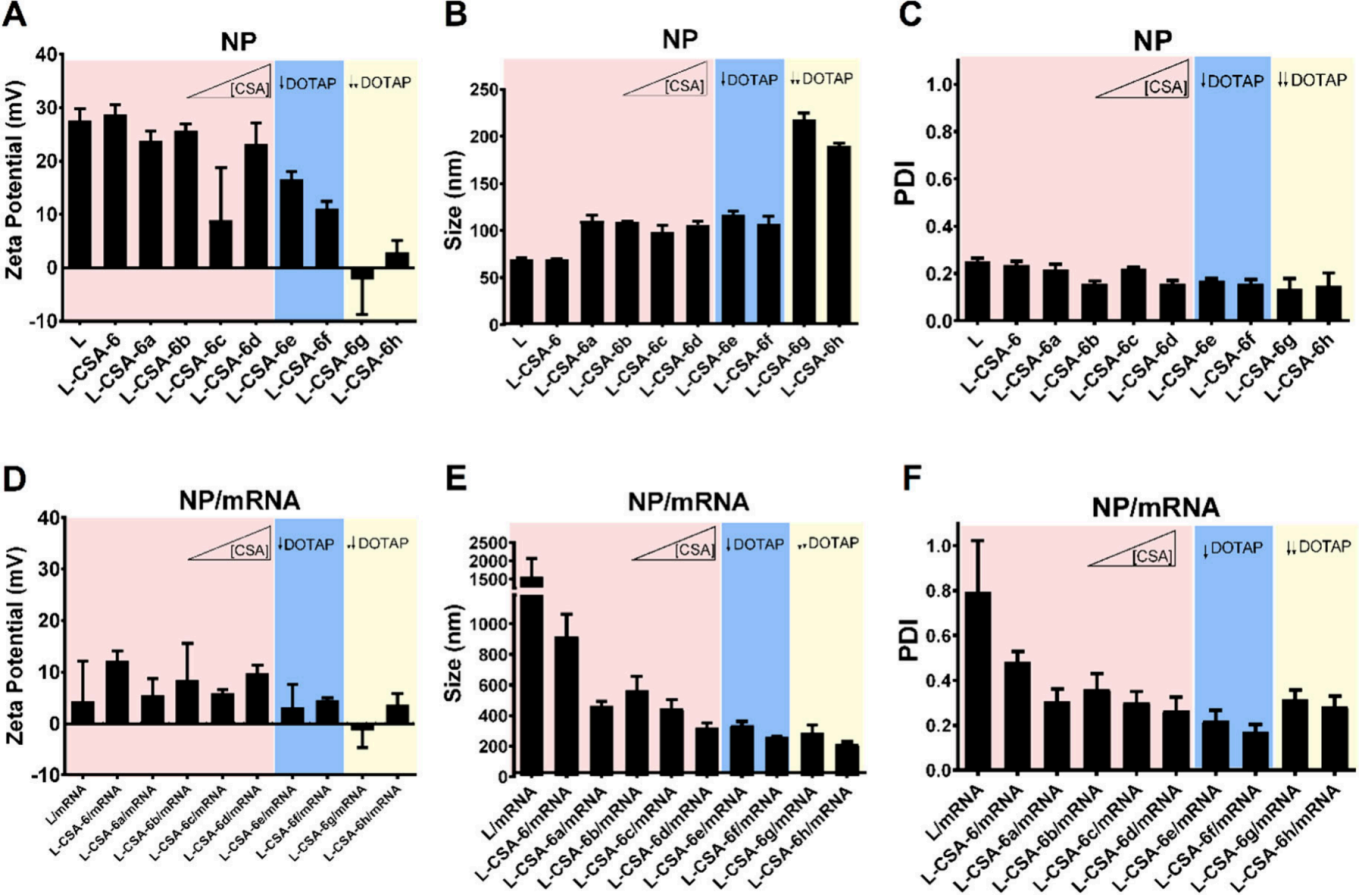
Comparison of NPs and NPs complexed with mRNAs. Measurement of (A, D) zeta potential, (B, E) size via DLS, and (C, F) PDI of (A–C) NPs and (D–F) NPs complexed with mRNA at an 8:1 (NP/mRNA) weight ratio. The color rose increase indicates the effect of increasing the concentration of CSA with a 1.0 mg/mL DOTAP concentration, whereas blue and yellow indicate the effects of decreasing the concentration of DOTAP to 0.5 and 0.1 mg/mL, respectively. The concentrations of CSA-6 in the NPs are presented in Table 1, and details about preparation are provided in Tables S1 and S2.

A significant reduction in zeta potential was observed with decreasing DOTAP concentration in the NP formulations (Figure 4). When the negatively charged mRNA bound to the NPs, a reduction in the surface charge was observed for all the NPs, except for L-CSA-6g-h and L-CSA-6c (Figures 4A and 4D). Moreover, agarose gel electrophoresis confirmed complexation with all the NPs, including L-CSA-6g-h and L-CSA-6c. (Figure S2). These findings suggested that even with a reduced DOTAP concentration, CSA significantly contributes to complexation with mRNA. Additionally, the DLS data indicated that an increase in the CSA concentration coupled with a decrease in the DOTAP concentration led to the formation of larger NPs (Figure 4B). However, this trend was reversed when the NPs were complexed with mRNAs (Figure 4E and F).

**Figure 5:**
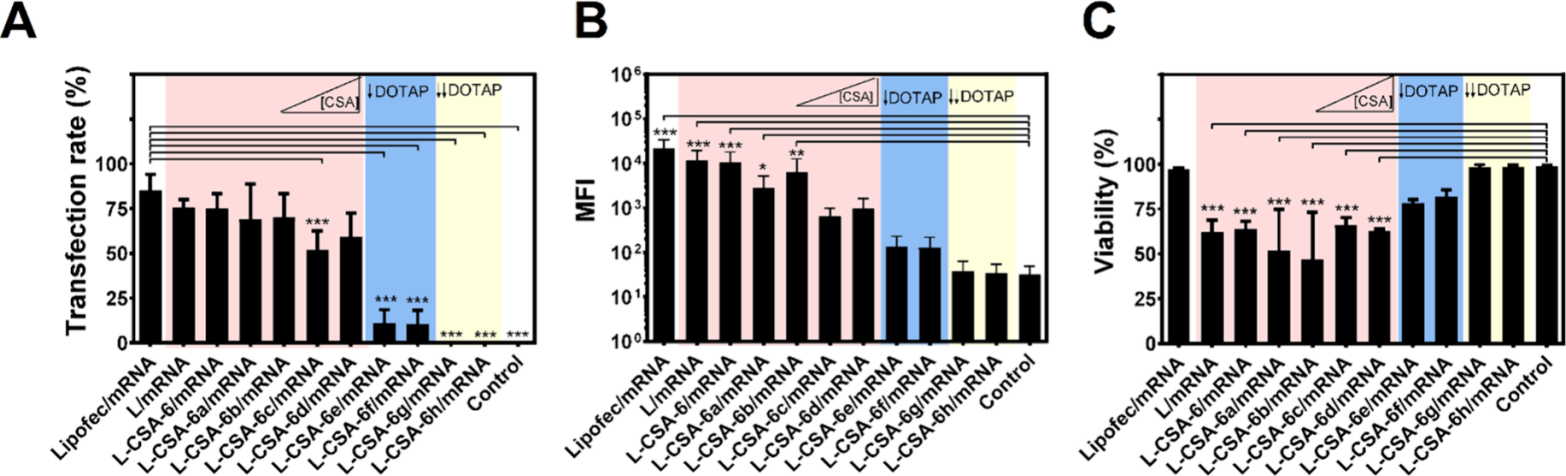
Effect of chitosomes on mRNA delivery. HEK293T cells were incubated with lipofectamine, L, and L-CSA-6(a-h) at an 8:1 ratio, and 250 ng of mRNA per well was added. The efficiency was evaluated at 48 h post-transfection. The (A) transfection rate, (B) MFI, and (C) cell viability were assessed by flow cytometry. The color rose indicates the effect of increasing the concentration of CSA with 1.0 mg/mL DOTAP, whereas blue and yellow indicate the effects of decreasing the concentrations of DOTAP to 0.5 and 0.1 mg/mL, respectively. The concentrations of CSA-6 in the NPs are presented in Table 1, and details about preparation are provided in Tables S1 and S2. Two-way ANOVA was used to compare the differences between the Lipofec/mRNA group and the other groups (A), as well as between the control group and the other groups (B, C). *p < 0.05, **p < 0.01, and ***p < 0.001.

Given that these NPs effectively formed complexes with mRNAs at an 8:1 ratio, demonstrated an appropriate size and PDI, and the majority exhibited positive zeta potential, the efficiency of these NPs in the transfection of HEK293T cells with 250 ng of mRNA per well was evaluated at 48 h post-transfection (Figure 5 and Figure S3).

The transfection efficiency of both L- and chitosomes L-CSA-6a-b was comparable to that of Lipofectamine, ranging from 57.7% to 85.2% of efficiency. Compared with L-CSA-6a-b, the chitosomes L-CSA-6c-d had slightly lower transfection rates, and chitosomes L-CSA-6e-h much lower transfection rates. Nontransfected cells (control) did not show any sign of transfection (Figure 5A). The variation in MFI among different NPs, corresponding to variations in the transfection rate, indicated that transfection occurred uniformly among the cells. These data indicated that the best ratio of CSA-6:DOPE:DOTAP (in mg/mL) for transfection is 0.5–1:1:1.

A positive correlation between cell viability and reduced DOTAP concentrations was evident, even in formulations with increased levels of CSA-6, indicating that CSA-6 does not significantly contribute to cellular toxicity. The present data showed that the efficiency of transfection is closely related to the amount of DOTAP present; lower DOTAP levels correspond to reduced transfection efficiency. This observation aligns with previous research suggesting that the effectiveness of cationic lipids, such as DOTAP, in enhancing transfection is largely due to their interaction with endosomal phospholipids and their ability to facilitate or drive the phase transition from lamellar to hexagonal structures, aiding in efficient endosomal escape.^36,37^ Furthermore, the morphology of the NP/mRNA complexes changes with increasing ratios of DOPE to DOTAP, potentially leading to diminished transfection efficiency.^38^

These findings also provide valuable insights into the role of CSA in the chitosome formulations under investigation. CSA plays an important role in the complexation of mRNA and contributes to the compaction of the resulting complex (Figure 4E). However, elevated concentrations of CSA lead to the formation of highly stable NP/mRNA complexes that are harder to dissociate, which in turn decreases protein expression levels (Figure 5B). As a result, endosomal escape is dependent on the DOTAP concentration, as the transfection efficiency decreases with DOTAP concentration decreases (Figure 5A).

### 3.3 Transfection Efficiency and the Optimal Ratio of Chitosomes/mRNAs Are Dependent on Cell Type

The efficiency with which exogenous genetic material is delivered into cells can vary significantly due to differences in cell membrane composition, size, shape, and rate of multiplication. This variability makes transfection more challenging in certain cell types. Understanding these differences is crucial for ensuring the reproducibility of results and for evaluating the effectiveness of nanocarriers across various biological contexts.

The initial experiments employed the HEK293T cell line, an epithelial-like cell line derived from the kidney of an aborted human female embryo. The HEK293T cell line is one of the most commonly used human cell lines for expressing recombinant proteins, primarily because of its rapid growth rate and ease of culture.^39^ To evaluate the transfection efficiency of chitosome/mRNA in diverse cell types, the following two cell lines commonly used in biomedical research were utilized: HeLa, the oldest human tumor cell line; and NIH-3T3, a widely used murine fibroblast line.^40−43^

**Figure 6:**
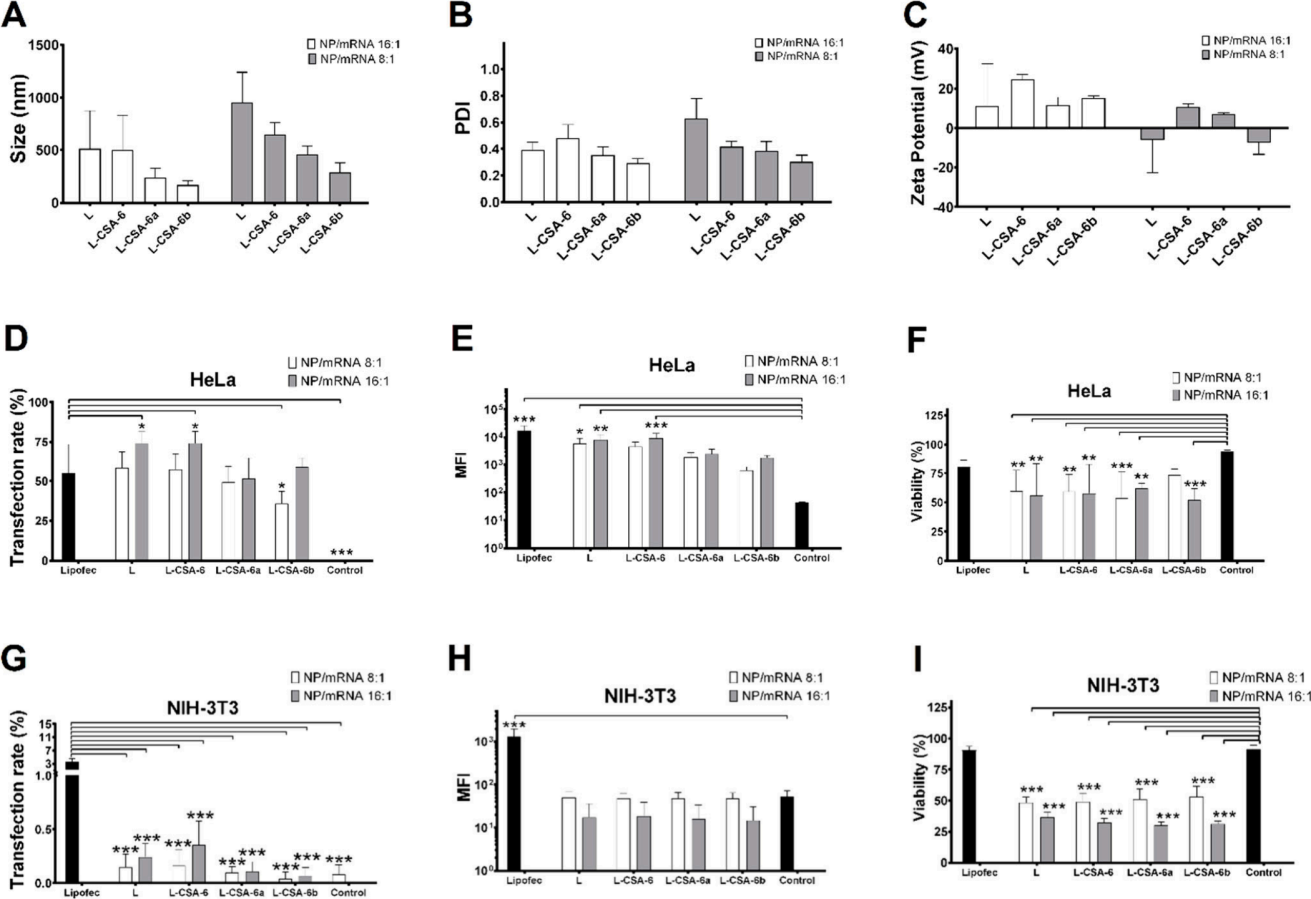
Particle characterization and transfection efficiency of NP/mRNA in HeLa and NIH-3T3 cells. The complexation of L, L-CSA-6, L-CSA-6a and L-CSA-6b with mRNAs at 8:1 and 16:1 (NP:mRNA) ratios was characterized by (A) size, (B) PDI, and (C) zeta potential. (D–F) HeLa cells and (G–I) NIH-3T3 cells were incubated with NPs/mRNA at 8:1 and 16:1 weight ratio, with 150 ng of mRNA per well, and the efficiency was evaluated at 48 h post-transfection. (D, G) Transfection rate, (E, H) MFI, and (F, I) cell viability were assessed by flow cytometry. One-way ANOVA was used to compare the differences between Lipofec/mRNA and the other groups in terms of the transfection rate (D, G) or between the control group and the other groups in terms of the MFI and viability (E, F, H, and I). *p < 0.05, **p < 0.01, and ***p < 0.001.

To test the different cell lines, 150 ng of mRNA was used because there was no significant difference in transfection efficiency or cell viability in comparison to 250 ng of mRNA in HEK293T cells (Figure S4). In addition, two NP/mRNA weight ratios (8:1 and 16:1) were used for transfection analysis. The results of particle characterization at a 16:1 ratio and transfection efficiency are presented in Figures 6 and S5.

The results of particle characterization at the 8:1 ratio revealed a significant difference in size only with respect to L, which was measured at 957 nm ±284 nm with 150 ng of mRNA (Figure S5). Notably, only the L-CSA-6b complex revealed a change in charge, exhibiting an increase in charge as the concentration increased. The observed negative zeta potentials of L and L-CSA-6b may be attributed to the conformational changes in the NP/mRNA complexes, indicating the greater presence of mRNAs outside of the vesicles. Thus, the distribution of charges on the nanoparticles becomes less uniform, possibly due to greater variability in the arrangement of mRNAs within the complexes. No significant changes in PDI were found across all the compositions (Figure S5).

All the NP/mRNA complexes at a 16:1 ratio exhibited a smaller size and greater charge than those at a 8:1 ratio with 150 ng of mRNA (Figure 6). The sizes of the L/mRNA and L-CSA-6/mRNA complexes, which were 506 nm, were comparable at a 16:1 ratio. Similarly, L-CSA-6a and L-CSA-6b exhibited no significant difference in size and had an approximate size of 202 nm. The PDI and zeta potential of the NP/mRNA complexes did not significantly vary across the samples.

The transfection efficiencies of L, L-CSA, L-CSAa, and L-CSAb were evaluated in HeLa and NIH-3T3 cells. In HeLa cells, Lipofectamine exhibited a transfection efficiency of 55.2% ± 18.3%, and the compositions L/mRNA (58.5% ± 10.1%), L-CSA-6/mRNA (57.4% ± 9.6%) and L-CSA-6a/mRNA (49.4% ± 9.7%) did not differ significantly from Lipofectamine. Only L-CSA-6b showed a lower transfection efficiency (35.8% ± 7.6%). Moreover, increasing the NP/mRNA ratio to 16:1 improved transfection rate for both chitosome and liposome formulations, reaching 74.2% ± 7.1% (L/mRNA), 74.3% ± 7.3% (L-CSA-6/mRNA), 51.5% ± 13.0% (L-CSA-6a/mRNA), and 58.9% ± 5.5% (L-CSA-6b/mRNA), highlighting the compositions L/mRNA and L-CSA-6/mRNA, which were significantly better than Lipofectamine in HeLa cells (Figures 6 and S6).

In NIH-3T3 cells, at 8:1 and 16:1 ratios, the complexes showed very low transfection efficiencies, and although some green fluorescent cells were present in transfection at 16:1 ratio (Figure S6), all compositions showed significantly lower efficiency than Lipofectamine (Figure 6G). These findings suggested limitations in the mRNA delivery of these formulations in NIH-3T3 cells, highlighting the need for further optimization for this cell type.

HeLa cells transfected with NP/mRNA at a 16:1 ratio had low viability as follows: approximately 56% for L/mRNA, 58% for L-CSA-6/mRNA, 62% for L-CSA-6a/mRNA, and 52% for L-CSA-6b/mRNA. Transfection at an 8:1 ratio enhanced cell viability in most compositions as follows: approximately 60% for L-CSA-6/mRNA, 59% for L-CSA-6/mRNA, 54% for L-CSA-6a/mRNA, and 73% for L-CSA-6b/mRNA.

Despite the low transfection efficiency in NIH-3T3 cells, the cell viability was also low. The cell viability after transfection with NP/mRNA was approximately 37% for L/mRNA, 32% for L-CSA-6/mRNA), 30% for L-CSA-6a/mRNA, and 31% for L-CSA-6b/mRNA at a 16:1 ratio, whereas at an 8:1 ratio it was 48%, 49%, 51%, and 53%, respectively, for the same complexes. Taken together, these results demonstrated that the DOTAP cationic lipid is significantly toxic at concentrations of 0.5–1.0 mg/mL and that the presence of CSA mitigates this toxicity. As previously discussed, flow cytometry analysis has limitations in distinguishing dead cells from permeabilized cells (Figure 3). While HEK293T cells recovered quickly after transfection, HeLa and NIH-3T3 cells did not recover within 48 h, suggesting that they may have required a longer recovery period. These findings underscore the importance of carefully optimizing the chitosome composition for specific cell types, taking into consideration factors, such as cell permeability and effective cytotoxicity.

Although liposomes and chitosomes showed promising results in HEK293T and HeLa cells, their transfection efficiency was notably lower in NIH-3T3 cells. Several factors may contribute to this discrepancy, one of the most significant being the distinct cellular composition of each cell line, which influences how nanocarriers interact with the cell membrane.^44,45^ Different membrane-associated proteins, such as clathrin, caveolin, and endophilin-A (EndoA), can affect nanoparticle uptake by enhancing or inhibiting specific endocytic pathways.^44,46,47^ The variability in the expression of these proteins across cell types may explain the reduced transfection efficiency observed in NIH-3T3 cells compared to HEK293T or HeLa cells.

Additionally, the lipid composition of the cell membrane plays a critical role in endocytosis.^48,49^ Lipid rafts and other membrane domains involved in vesicle formation and endocytic uptake can vary between cell lines, potentially leading to differences in how nanoparticles are internalized and processed. For example, lipid domains enriched with cholesterol and sphingolipids may favor one endocytic pathway over another, thereby affecting transfection efficiency depending on the specific nanocarrier used.^44,48−51^

Given these factors, further studies aimed at elucidating the mechanisms of cellular internalization—particularly through endocytic pathways—are essential for fully understanding the differences in transfection efficiency across different cell lines.

The use of NPs extends beyond *in vitro* transfection, as they are also utilized for gene therapy and genetic vaccines. Recent studies have identified DOTAP as a potent inducer of the innate immune system in animal cells. DOTAP increases the production of reactive oxygen species (ROS), which subsequently trigger apoptosis.^52^ Although this characteristic may be advantageous in vaccine development, it may not be suitable for therapeutic applications, particularly in the context of inflammatory diseases. Given the demonstrated properties of chitosomes, they present a compelling platform for *in vivo* experiments.

## 4 Conclusions

In conclusion, the findings of this study suggest that chitosomes may be an effective and viable alternative to traditional transfection reagents for mRNA delivery. The incorporation of CSA into lipid formulations enhances the stability of chitosomes and promotes efficient complexation with mRNA and high transfection efficiency in HeLa and HEK293T cells. However, it is important to note that increased complex stability may hinder mRNA release, potentially resulting in reduced transfection efficiency. Moreover, the addition of CSA may play a critical role in reducing the cytotoxicity associated with DOTAP. Finding the right balance between CSA and DOTAP is highly important for creating mRNA nanoparticles that have high transfection efficiency and reduced cytotoxicity. Notably, the transfection efficiency of chitosomes may be limited in certain cell types, such as NIH-3T3 cells. Further studies are needed to understand the factors that limit the transfection efficiency of chitosomes in these cell types and to develop strategies to improve their efficacy. Overall, these findings provide important insights into the role of CSA in chitosome formulations and into the function of each component in a hybrid system, which is crucial for developing alternative nonviral nucleic acid delivery systems.

## Supporting Information Available

Details about the chitosome preparation, chitosome concentration, flow cytometry data analysis, transfection efficiency, and mRNA complexation (PDF)
