# Supplementary material for: Efficacy of Chitosan-N-Arginine Chitosomes in mRNA Delivery and Cell Viability Enhancement
Source: ACS Appl Bio Mater. 2024 Nov 19;7(12):8261–71. doi: 10.1021/acsabm.4c00983 (PMC11653394; doi:10.1021/acsabm.4c00983)
Supplement: Supplementary file 1 — mt4c00983_si_001.pdf [file mt4c00983_si_001.pdf]

SUPPORTING INFORMATION

Efficacy of Chitosan-N-Arginine Chitosomes in mRNA Delivery and Cell Viability Enhancement

Bianca B. M. Garcia[a,b], Stefania Douka[b], Omar Mertins[a], Enrico Mastrobattista[b], Sang W. Han*[a]

[a]Department of Biophysics, Paulista School of Medicine, Federal University of São Paulo, 04023-062 São Paulo, Brazil

[b]Department of Pharmaceutics, Faculty of Science, Utrecht University, Universiteitsweg 99, 3584 CG Utrecht, The Netherlands

Corresponding Author

*E-mail: sang.han@unifesp.br

ORCID: 0000-0002-4953-7680

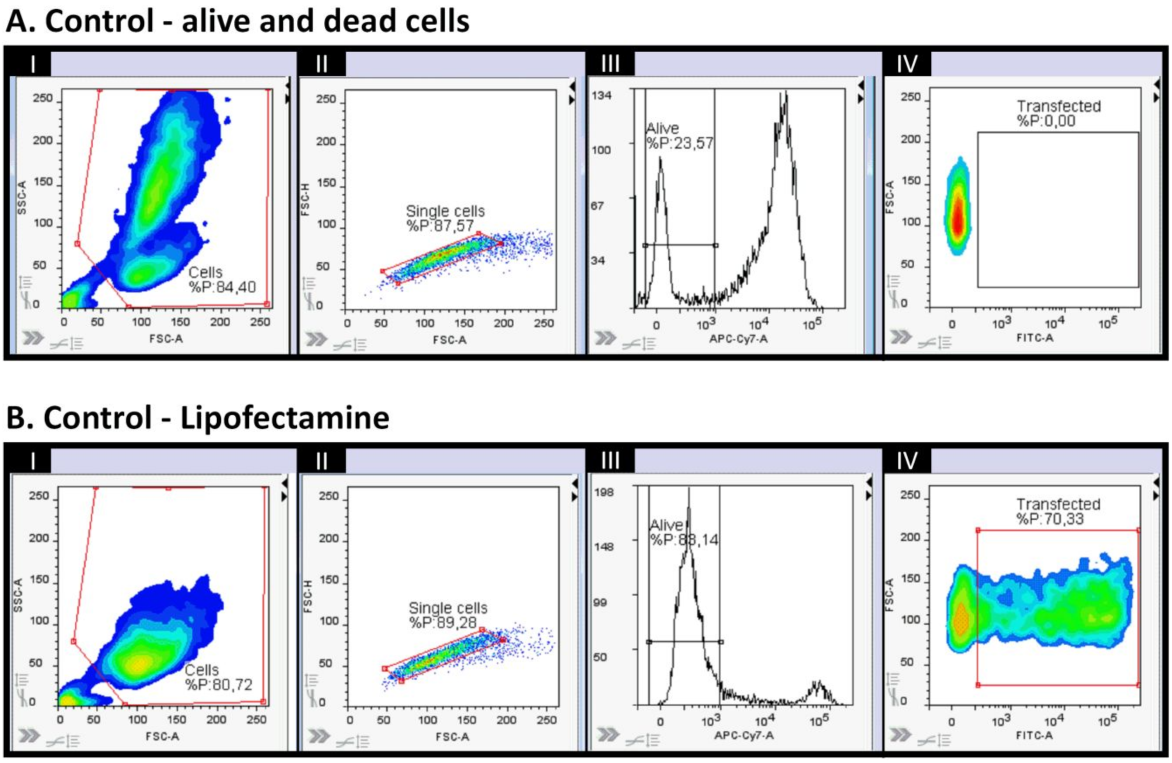

Figure S1: Gating strategy applied in flow cytometry data analysis. **(A)** To separate alive population, non-transfected cells were mixed with non-transfected dead cells, that were killed by heat shock. **(B)** Alive and transfected population were selected

using cells transfected with lipofectamine. **(I)** Low FSC events were eliminated by gating the homogeneous cell population. **(II)** Single cells were selected by gating of homogeneous cell population from FSC-A over FSC-H. **(III)** Alive cells population were selected by APC-Cy7 fluorescence. **(IV)** Transfected cells population were selected by FITC fluorescence.

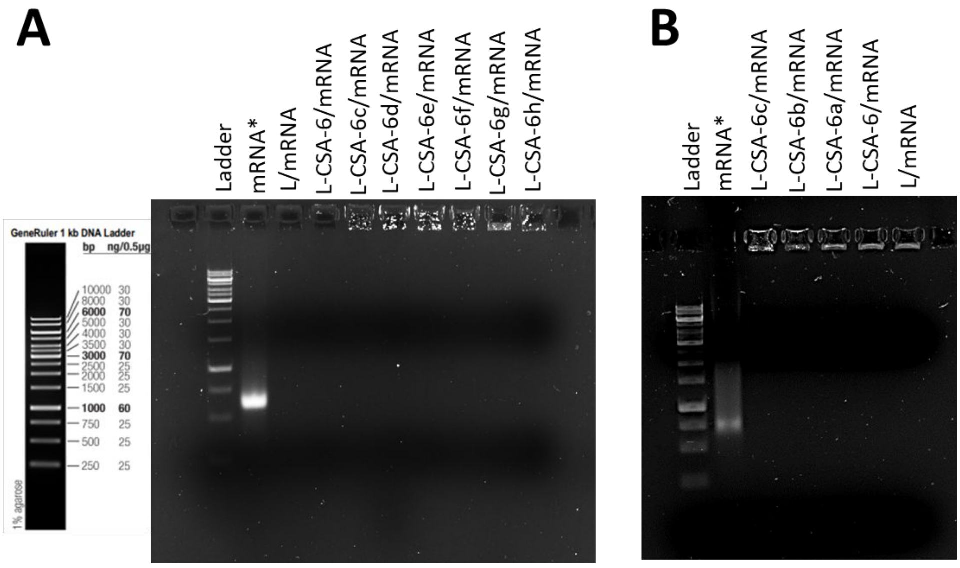

Figure S2: Investigation of NP/mRNA complexation. Liposome and chitosomes **(A)** L-CSA6(c-h) and **(B)** L-CSA6(a-c) were formulated with mRNA in a 8:1 ratio and were analyzed by gel electrophoresis.

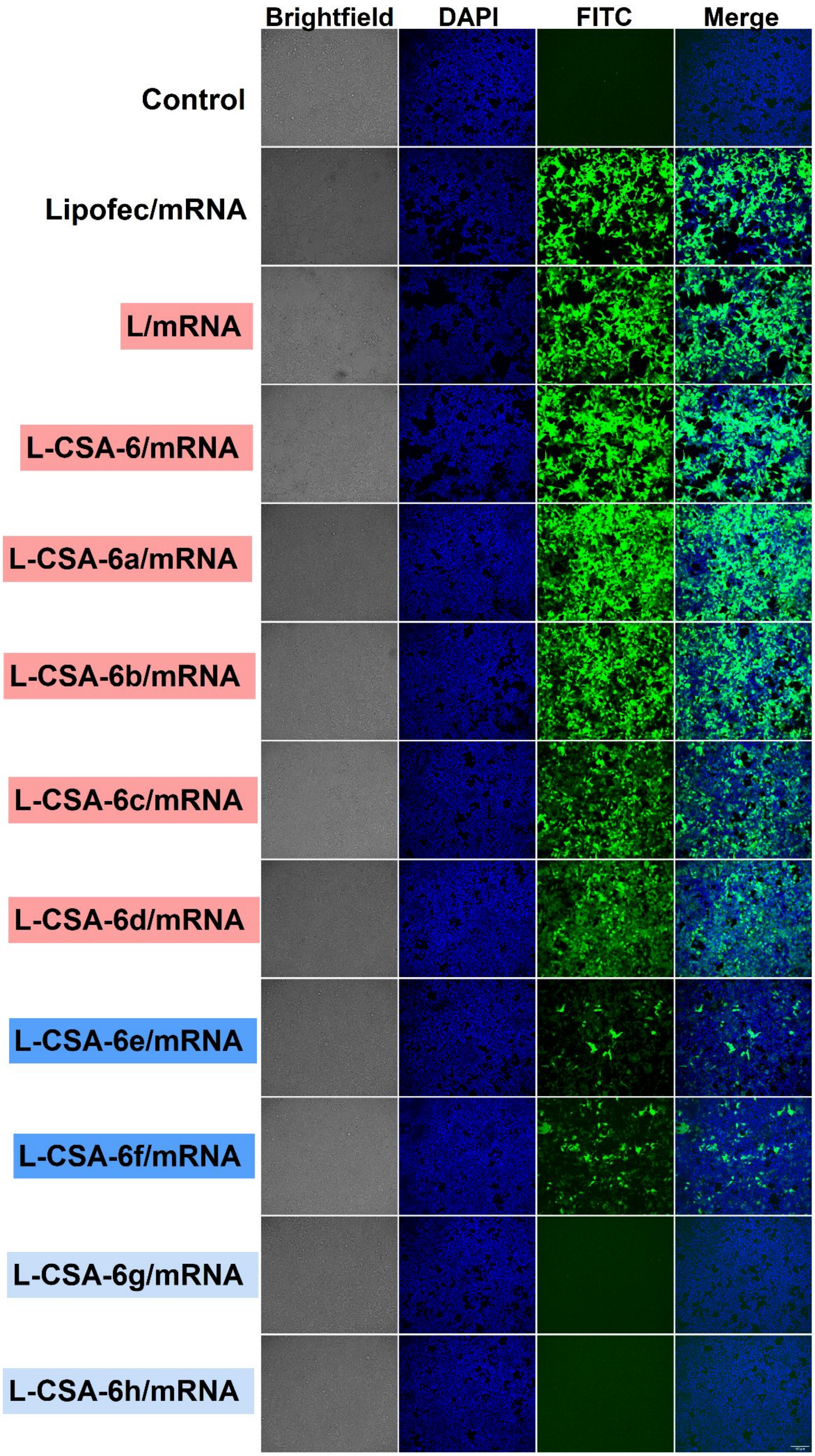
Brightfield
DAPI
FITC
Merge
Control
Lipofec/mRNA
L/mRNA
L-CSA-6/mRNA
L-CSA-6a/mRNA
L-CSA-6b/mRNA
L-CSA-6c/mRNA
L-CSA-6d/mRNA
L-CSA-6e/mRNA
L-CSA-6f/mRNA
L-CSA-6g/mRNA
L-CSA-6h/mRNA

Figure S3: Fluorescent microscopy images of the effect of chitosomes composition on mRNA transfection efficiency. HEK293T cells were incubated with lipofectamine, L, and L-CSA-6a-h at an 8:1 ratio, maintaining 250 ng of mRNA per well, and efficiency was evaluated 48 h post-transfection.

Aiming to further optimize cell viability post transfection, we examined the use of chitosomes at an 8:1 ratio, but at a total mRNA concentration of 150 ng instead of 250 ng per well. Transfection analysis was performed 48 h after incubating NP/mRNA complexes with the cells, as shown in Figure S4. In this experiment, we used NPs L, L-CSA-6, L-CSA-6a, and L-CSA-6b. The transfection efficiency of L and chitosomes L-CSA-6(a-b) was similar, ranging from 60.0% to 86.4%.

Only L-CSA-6 exhibited a significant difference compared to Lipofectamine. The cell viability of L and chitosomes L-CSA-6a-b did not differ significantly among them. However, they differed significantly from both Lipofectamine and non-transfected cells (control), ranging from 61.4% to 74.2%. Since there was no significant difference in transfection efficiency and cell viability between the two mRNA quantities, we used the lower dose of 150 ng for further testing in other cell lines.

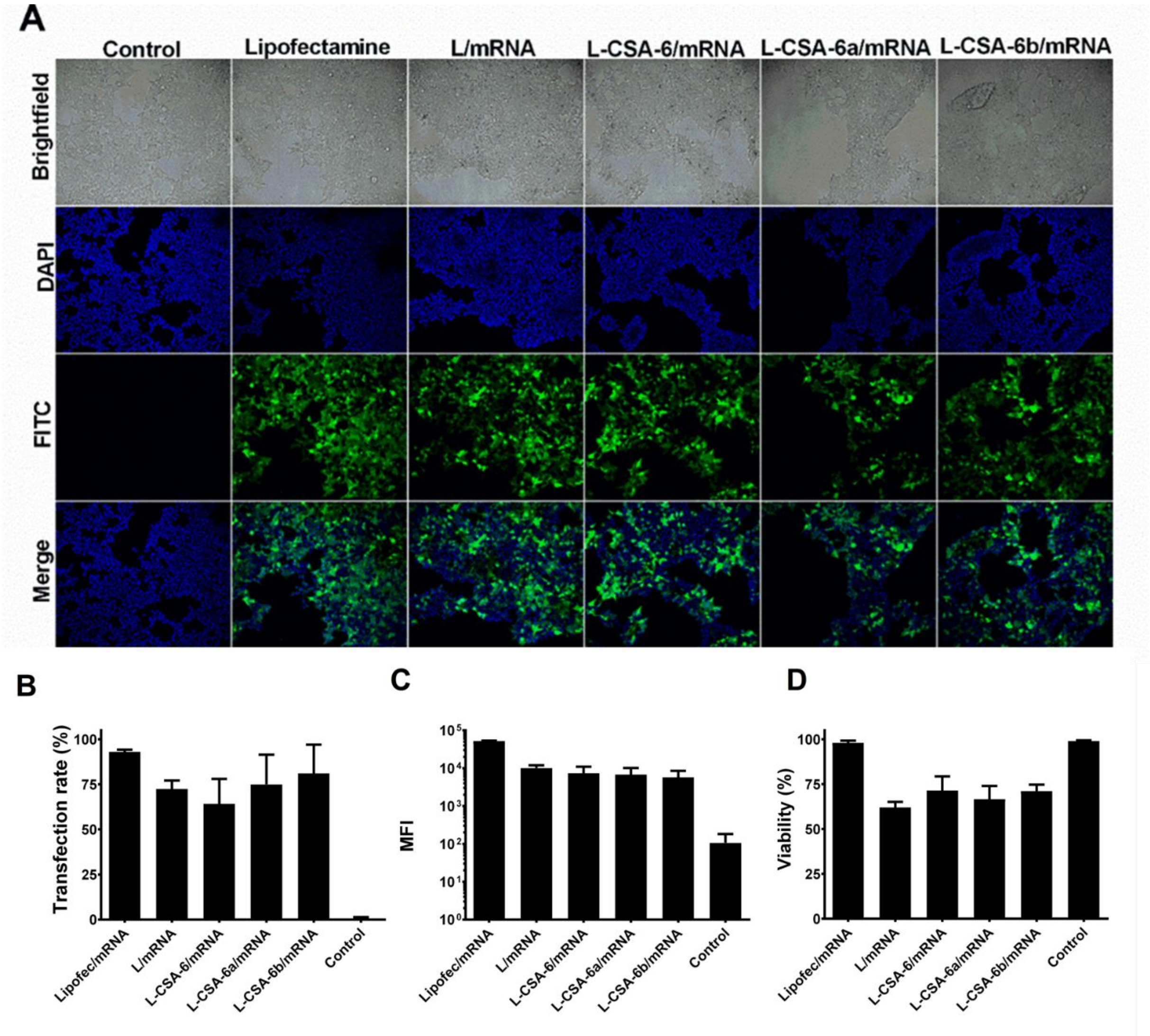

Figure S4: Transfection efficiency of optimized protocol in HEK293T cells at 150 ng mRNA concentration. HEK293T cells (12,500 cells/well in a 96-well plate) were incubated with lipofectamine, L, and L-CSA-6(a-b) at 8:1 ratio, and efficiency was evaluated 48 h post-transfection. Results of **(A)** transfection efficiency were assessed qualitatively by fluorescence microscopy images, and evaluation of **(B)** transfection rate, **(C)** MFI and **(D)** cell viability by flow cytometry.

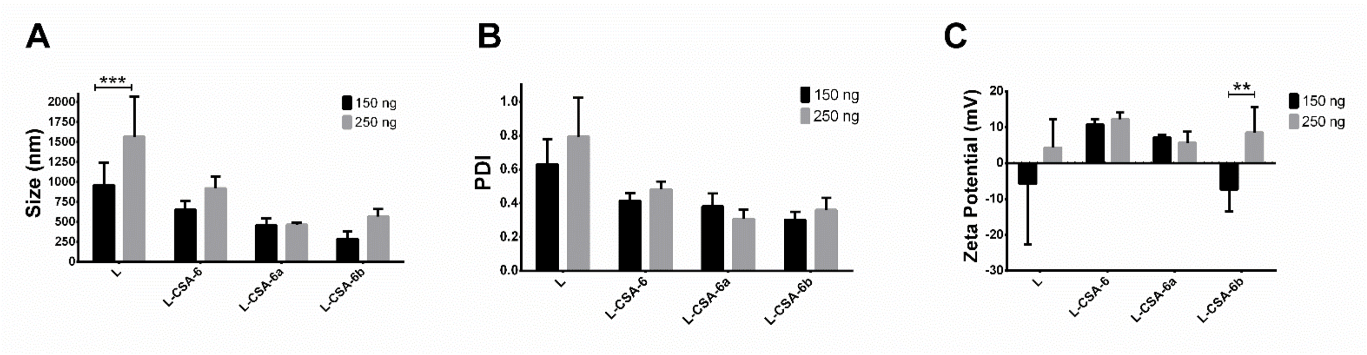

Figure S5: NP/mRNA particle characterization. Comparison by **(A)** Size, **(B)** PDI, and **(C)** Zeta potential of nanoparticles L, L-CSA-6, L-CSA-6a, and L-CSA-6b complexed with mRNA at 8:1 weight ratio using 150 nm or 250 ng of mRNA. 2-way ANOVA was used to compare the different mRNA doses. * $p < 0.05$.

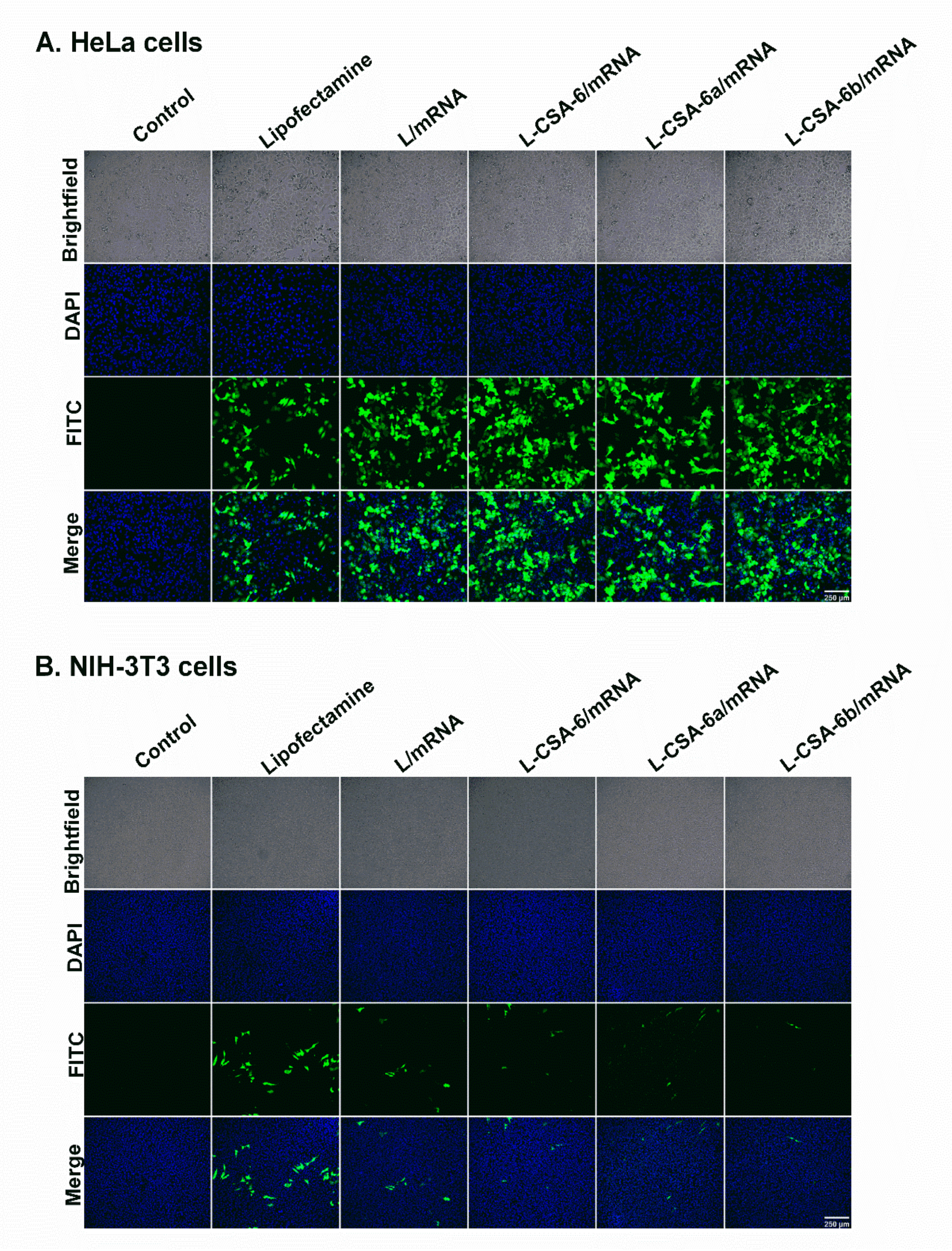

Figure S6: Panel of transfection efficiency of chitosome/mRNA formulated with the optimized protocol in **(A)** HeLa and **(B)** NIH-3T3 cells. Cells were incubated with lipofectamine, L, and L-CSA-6a-b at 16:1 ratio (keeping 150 ng of mRNA per well), and efficiency was evaluated 48 h post-transfection. The results of transfection efficiency

were assessed qualitatively by fluorescence microscopy images. Scale bar represents 250µm.

Table S1: Amounts of DOPE, DOTAP, and CSA used for particle preparation. After solvent evaporation, NPs were resuspended in 5 mL of H_2O nuclease-free.

NP name	Preparation DOPE* (10 mg.mL^{-1})	DOTAP* (10 mg.mL^{-1})	CSA** (1 mg.mL^{-1})
**L**	500 µL	500 µL	-
**L-CSA-3**	500 µL	500 µL	200 µL (CSA-3)
**L-CSA-6**	500 µL	500 µL	200 µL (CSA-6)

* Prepared in chloroform. **Prepared in MilliQ Water

Table S2: Amounts of DOPE, DOTAP, and CSA used for particle preparation . After solvent evaporation, new NPs were resuspended in 1 mL of H_2O nuclease-free.

NP name	Preparation DOPE* (10 mg.mL^{-1})	DOTAP* (10 mg.mL^{-1})	CSA-6** (10 mg.mL^{-1})
**L-CSA-6a**	100 µL	100 µL	50 µL
**L-CSA-6b**	100 µL	100 µL	100 µL
**L-CSA-6c**	100 µL	100 µL	200 µL
**L-CSA-6d**	100 µL	100 µL	400 µL
**L-CSA-6e**	100 µL	50 µL	200 µL
**L-CSA-6f**	100 µL	50 µL	400 µL
**L-CSA-6g**	100 µL	10 µL	200 µL
**L-CSA-6h**	100 µL	10 µL	400 µL

* Prepared in chloroform. **Prepared in MilliQ Water

Table S3: Volume of NP used to complex 1 µg of mRNA at different NP/mRNA ratios.

NP	[DOPE:DOTAP] (µg.µL^{-1})	NP volume (µL) to complex 1 µg of mRNA			
		2:1	4:1	8:1	16:1
**L**	2	1	2	4	8
**L-CSA-3**	2	1	2	4	8
**L-CSA-6**	2	1	2	4	8
**L-CSA-6a**	2	1	2	4	8
**L-CSA-6b**	2	1	2	4	8
**L-CSA-6c**	2	1	2	4	8
**L-CSA-6d**	2	1	2	4	8
**L-CSA-6e**	1.5	1.3	2.7	5.3	10.7
**L-CSA-6f**	1.5	1.3	2.7	5.3	10.7
**L-CSA-6g**	1.1	1.8	3.6	7.3	14.5
**L-CSA-6h**	1.1	1.8	3.6	7.3	14.5
